# Vanadium and biomarkers of inflammation and oxidative stress in diabetes: A systematic review of animal studies

**DOI:** 10.34172/hpp.2022.16

**Published:** 2022-08-20

**Authors:** Faezeh Ghalichi, Alireza Ostadrahimi, Maryam Saghafi-Asl

**Affiliations:** ^1^Student Research Committee, Tabriz University of Medical Sciences, Tabriz, Iran; ^2^Department of Clinical Nutrition, Faculty of Nutrition and Food Sciences, Tabriz University of Medical Sciences, Tabriz, Iran; ^3^Nutrition Research Center, Drug Applied Research Center, Department of Clinical Nutrition, Faculty of Nutrition & Food Sciences, Tabriz University of Medical Sciences, Tabriz, Iran

**Keywords:** Systematic review, Animals, Diabetes mellitus, Inflammation, Oxidative stress

## Abstract

**Background:** Oxidative stress has a significant role in the commencement and development of hyperglycemia. Vanadium, as a transitional metal with redox properties, enters the redox process, produces free radicals, and distracts the pro-antioxidant balance. The present animal systematic review aimed to assess the effect of vanadium supplementation on inflammation and oxidative stress biomarkers in diabetes-induced animals.

**Methods:** A systematic search was conducted using the PubMed, Scopus, and web of science databases from 1990 to 2021, according to the inclusion and exclusion criteria. The search strategy was based on the guidelines for systematic review of animal experiments and Preferred Reporting Items for Systematic Reviews (PRISMA). Criteria for eligibility were animal-based studies, evaluating the therapeutic effects of vanadium on inflammatory and oxidative stress biomarkers in diabetes. The Systematic Review Centre for Laboratory Animal Experimentation (SYRCLE) tool was used for assessing the methodological quality of included studies.

**Results:** In the present study, 341 articles were evaluated out of which 42 studies were eligible for inclusion. The majority of the studies confirmed the advantageous properties of vanadium on inflammatory and oxidative stress biomarkers. A minor risk of bias was reported, based on the SYRCLE’s tool.

**Conclusion:** According to the findings, well-designed clinical trials are warranted to assess the long-lasting effects of various vanadium compounds on inflammatory and oxidative stress biomarkers.

## Introduction

 Type 2 diabetes mellitus (T2DM), represents nearly 95% of all cases of DM and is characterized by insulin resistance or a decline in β-cells’ ability to secrete insulin.^1^ In chronic hyperglycemia, glucose auto-oxidation leads to abundant production of oxygen-free radicals in the mitochondria due to major oxygen usage, high redox reactions, mitochondrial-fission state, and failure of the antioxidant defense system.^2-4^ Nevertheless, oxidative stress has a significant role in the commencement and ongoing of hyperglycemia, as well. In general, the inequity of reactive oxygen species (ROS) production and elimination is described as oxidative stress.^1^ ROS production leads to the impairment of nuclear deoxyribonucleic acid (DNA). Additionally, it stimulates nuclear poly (ADP-ribose) polymerase (PARP), prevents glyceraldehyde-3-phosphate dehydrogenase (GAPDH) activity, and shunts primary glycolytic substrates into pathogenic signaling pathways via the activation of I) the polyol, II) Protein kinase C (PKC), and III) glycation end-products (AGE) pathways.^5^

 The signaling pathways mentioned above augment ROS formation and stimulate inflammation. The polyol pathway intensifies ROS production using nicotinamide adenine dinucleotide phosphate (NADPH) and glutathione (GSH), and aggregating consequent nicotinamide adenine dinucleotide (NADH) oxidation. In addition, hyperglycemia reinforces inflammatory cytokines, including tumor necrosis factor-alpha (TNF-α) and nuclear factor kappa-light-chain-enhancer of activated B-cells (NF-κB) expression. Inhibition of GAPDH leads to dihydroxyacetone phosphate construction, as well as PKC and AGE increase. Such events consequently induce NADPH oxidase and the expression of inflammatory factors and decline endothelial nitric oxide synthase stimulation. Also, PKC stimulates insulin resistance via preventing downstream expression of phosphatidylinositol 3-kinase (PI3K)/protein kinase B (Akt) signaling pathway (PI3K-Akt).^4,5^

 Cellular ROS concentration is detected by the production and clearance rate of ROS. Antioxidant enzymes such as superoxide dismutase (SOD), catalase (CAT), glutathione reductase (GR), and glutathione peroxidase (GPx) are substrates that scavenge free radicals or inhibit their conversion to toxic derivatives.^6^ Thus, modulating these enzymes protect the cellular antioxidant system from oxidative stress.

 Since 1970s, the insulin-mimetic or insulin-enhancing properties of vanadium have been discussed and it has been considered as a therapeutic agent against diabetes.^7^ Vanadium, as a transitional metal with redox properties, enters the redox process, produces free radicals, and distracts the pro-antioxidant balance in the cell. Vanadium is a scavenger of superoxide radicals, and declines antioxidant enzymes such as SOD, GPx, CAT, and GR in erythrocytes,^8-11^ liver,^12-22^ kidneys,^14,19,20,22^ heart,^22^ brain,^22,23^ pancreas^16,20,24^ and testes^25^ of rats. Based on the results of multiple studies, vanadium complexes increase the action of GPx and demolish the effect of ROS in diabetic-induced rats.^6,8,22^

 Vanadium prevents protein tyrosine phosphatase activity and helps glucose transporter 4 translocation.^26^ Redox regulation inhibits PTP-1B activation. Due to the insulin-stimulating properties of NAD(P)H oxidase homolog Nox 4, it modulates H2O2 and plays an essential role in insulin signaling via modulating PTP-1B transcription.^27^ The complications of diabetes are directly associated with oxidative stress; hence, substrates reducing oxidative stress, are also beneficial for the complications of diabetes.^28^

 The beneficial effects of vanadium in declining hyperglycemia have already been reported in experimental and clinical trials.^29,30^ However, the objective of the current animal-based systematic review was to put together experimental evidence to present a thorough assessment of the effects of vanadium administration on inflammatory and oxidative stress biomarkers in diabetes-induced animals.

## Material and Methods

###  Search strategy

 The Preferred Reporting Items for Systematic Reviews (PRISMA) was implicated in this systematic review.^31^ PubMed, Scopus, and Web of Science databases were used for searching animal-based studies evaluating the effect of vanadium administration on inflammatory and oxidative stress biomarkers among diabetic animals from 1990 to 2021 (Table 1).^32-34^ Language restriction was not considered during the search strategy.

**Table 1:** Search strategy according to database filters

**Database**	**Search items**
PubMed	(("Vanadium"[Mesh] OR "Vanadium Compounds"[Mesh]) OR (vanadium[Title/Abstract])) AND ((((((("Diabetes Mellitus, Type 2"[Mesh]) OR "Obesity"[Mesh]) OR "Glucose Intolerance"[Mesh]) OR ( "Diabetes Mellitus"[Mesh] OR "Diabetes, Gestational"[Mesh] OR "Diabetes Mellitus, Type 1"[Mesh] OR "Diabetes Mellitus, Experimental"[Mesh] )) OR "Insulin"[Mesh]) OR "Glycated Hemoglobin A"[Mesh]) OR ((((((((Diabetes[Title/Abstract]) OR (obesity[Title/Abstract])) OR ("glucose intolerance"[Title/Abstract])) OR (Insulin[Title/Abstract])) OR ("Glycated hemoglobin A"[Title/Abstract])) OR (HbA1c[Title/Abstract])) OR (prediabetes[Title/Abstract])) OR (overweight[Title/Abstract])))
Scopus	((TITLE-ABS-KEY(Vanadium))) AND ((TITLE-ABS-KEY(Diabetes) OR TITLE-ABS-KEY (Obesity) OR TITLE-ABS-KEY (Overweight) OR TITLE-ABS-KEY (“Glucose Intolerance”) OR TITLE-ABS-KEY (Insulin) OR TITLE-ABS-KEY (“Glycated hemoglobin A”) OR TITLE-ABS-KEY (HBA1C) OR TITLE-ABS-KEY (Prediabetes)
Web of Science	((Vanadium)) AND ((Diabetes) OR (Obesity) OR (Overweight) OR (“Glucose Intolerance”) OR (Insulin) OR (“Glycated hemoglobin A”) OR (HBA1C) OR Prediabetes))

###  Inclusion criteria

 Eligible studies for including in this systematic review obeyed the **PICOS **criteria, as below^35^:

- **P**articipants: Diabetes-induced laboratory animals.
- **I**nterventions: Vanadium administration.
- **C**omparisons: Diabetic control animals, consuming a regular diet.
- **O**utcomes: Measuring inflammatory and oxidative stress biomarkers.
- **S**tudy design: Animal studies assessing the effect of vanadium administration in diabetic-induced animals.

###  Exclusion criteria 

 Studies assessing the effects of vanadium compounds on glycemic markers and lipid profile were excluded in this systematic review. Also, studies with invasive surgical procedures or certain diets were excluded.

###  Study selection

 Animal studies were screened individually by two investigators, according to the inclusion and exclusion criteria. At first, the titles and abstracts of selected studies were assessed; afterwards, the full texts were read carefully. In the end, the papers were monitored for final detection. Disagreements regarding selecting certain studies for inclusion were determined by discussion among investigators.

###  Data extraction

 A pre-standardized data extraction form was independently administered by two authors for extracting data. In the end, a third author was responsible for rechecking extracted data.

###  Assessment of methodological quality

 The Systematic Review Centre for Laboratory Animal Experimentation (SYRCLE)’s Risk of Bias (RoB) tool^36^ was used for evaluating the methodological quality and risk of bias of studies included, according to the Cochrane RoB tool. The SYRCLE’s RoB tool assesses 10 items, including selection bias, performance bias, detection bias, attrition bias, reporting bias, and other biases.

###  Outcomes

 Outcomes extracted from the included studies for evaluating the beneficial effects of vanadium were: (1) Inflammatory biomarkers such as TNF-α, interleukin 6 (Il-6), high-sensitivity C-reactive protein (hs-CRP); (2) oxidative stress biomarkers including GSH, SOD, CAT, GPx, glutathione S-transferase (GST), and GR.

## Results

###  Identification of relevant studies

**Figure 1:**
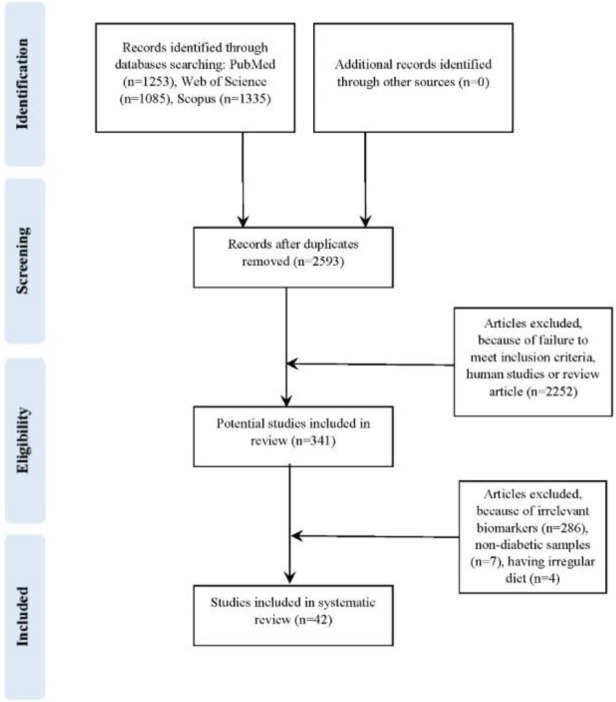
Flow diagram outlining included animal studies selection

 During the electronic search, 2593 potentially eligible studies were identified. Then, reviewing the title and abstract of studies resulted in the exclusion of 2252 studies, due to not fulfilling the inclusion criteria, or being randomized controlled trials or review articles. Afterward, 341 full-text papers were further reviewed. Eventually, 42 studies were eligible for inclusion. A flow diagram outlining the selection of final papers can be observed in Figure 1. Among the 42 studies included, 40 studies reported the beneficial therapeutic effects of vanadium on the enzymatic activity of inflammatory and oxidative stress biomarkers in diabetes-induced animal studies.

###  Characteristics of studies included in the analysis

 Table S1 summarizes the characteristics of animal studies included. The studies’ publication dates ranged from August 1990^37^ to November 2021.^38^ In 24 out of the included studies,^12,16-18,20,21,39-56^ randomization was reported. Sample size was from 20^57^ to 90^2^animals. Streptozotocin^2,12-14,16-21,37-42,44,45,48-61^ or alloxan monohydrate^6,8,11,22,24,46,47,62,63^ wereused for inducing diabetes, except for one study^43^ that used high-fat low-sucrose diet for inducing insulin resistance. All of the studies were accomplished on rodents. In 8 studies,^38,40,41,44,58,61^ Sprague-Dawley rats were used, while in the rest, Swiss Albino of Wistar strain rats were investigated. Additionally, 3 studies^2,16,43^ were on mice species. Rat’s mean weight was 175 g and mice’s mean weight was 26.5 g. Sources of vanadium consumed for administration were vanadyl sulfate (20 studies),^2,12,17,18,21,39-42,44,48-51,53,54,57,58,60^ sodium orthovanadate (10 studies),^6,8,11,19,22,37,46,62-64^ oxovanadium (IV) complex (1 study),^2^ vanadium pentoxide (2 studies),^20^ Na[(O2)2(2,2’-bpy)] • 8 H2O vanadium complexes (1 study),^13^ vanadyl(IV)-ascorbate (VOAsc) complex (1 study),^43^ dioxidovanadium cis-[VO2(obz)py]) complex (1 study),^38^ NaVO3 (3 studies),^21,47^ vanadium-3-hydroxy flavone complex (1 study),^59^ macrocyclic binuclear oxovanadium (IV) complex (MBOV) (1 study),^14^ bis(maltolato)oxovanadium (IV) (2 studies),^16,45^ dioxidovanadium (1 study),^61^ vanadium citrate (1 study),^24^ V3dipic-Cl ^21^ and oxovanadium(IV) chelate [VOL] (1 study).^51^ Method of administration was either by dissolving into drinking water,^6,8,11,16,17,19,21,22,24,37,40,41,44-47,57-59,62,63,65^ gavage,^12-14,18,20,38,39,42,43,48-54,61^ or intraperitoneal injection.^60^ Duration of the interventions ranged from 15 days up to 60 days. Measured outcomes were the enzymatic activity of inflammatory biomarkers such as TNF-α, Il-6, hs-CRP, caspase 3, as well as oxidative stress biomarkers including GSH, SOD, GPx, GST, and GR.

###  Quality assessment

**Figure 2:**
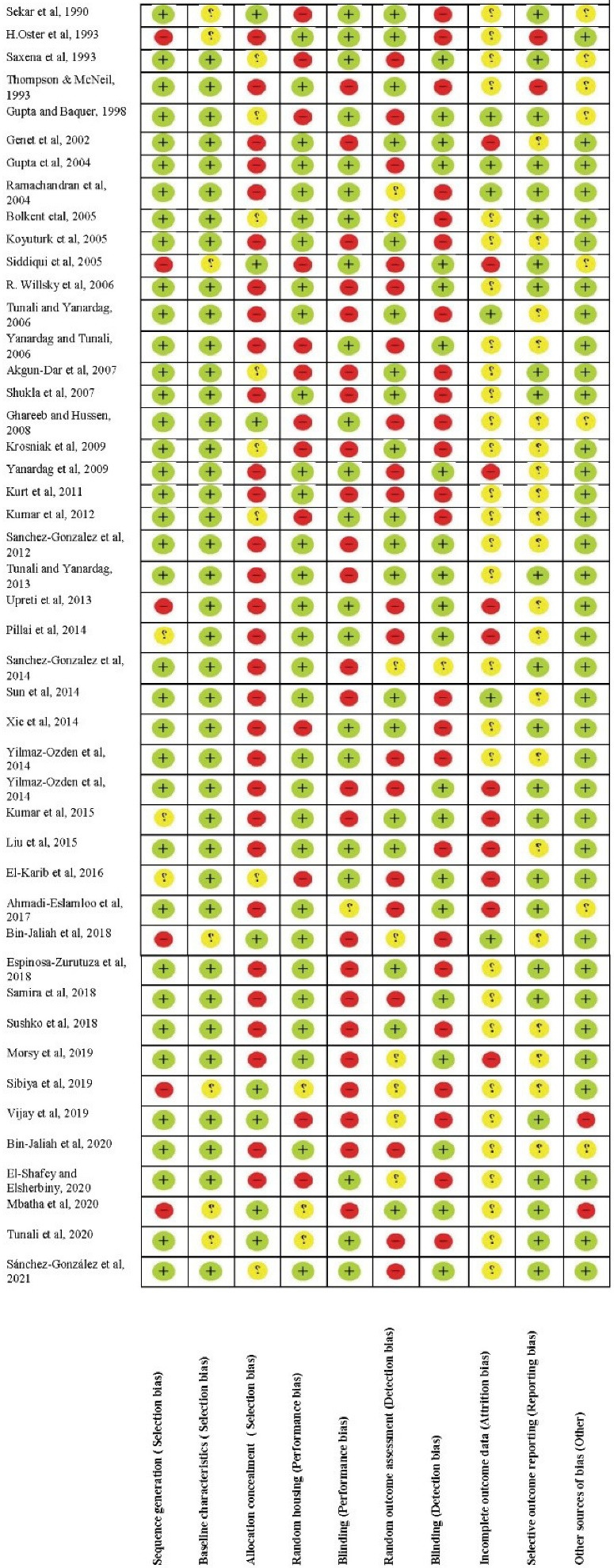
Risk of bias indicating studies’ quality assessment at an individual level. (+ ) Low risk of bias. (-) High risk of bias. (?) Unclear risk of bias

**Figure 3:**
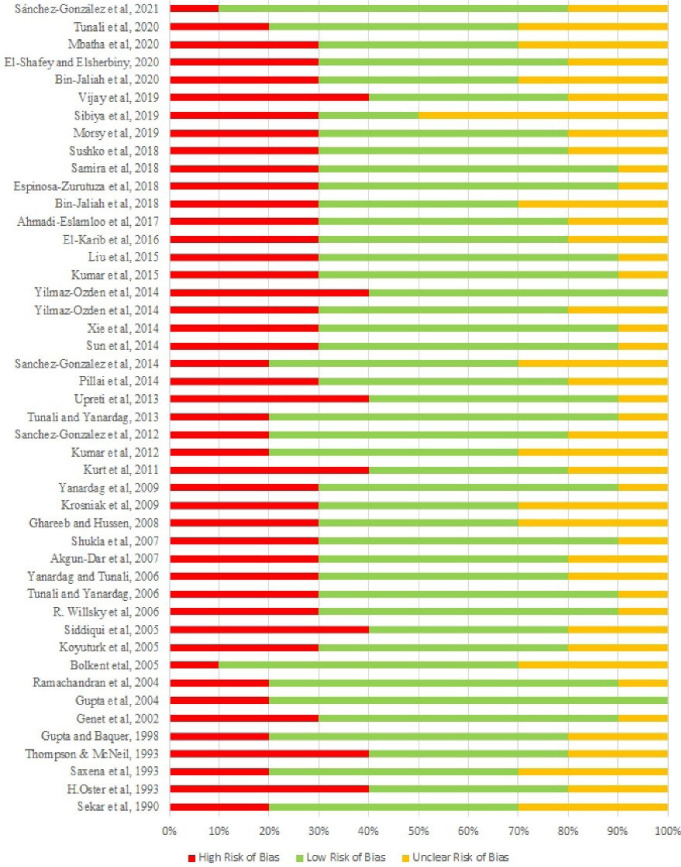
Risk of bias of included studies as percentages

 Randomization was reported in 24 of the included studies.^12,16-18,20,21,39-56^ Animals were similar in age and weight and were kept in controlled and similar conditions. Figure 2 illustrates the methodological quality assessment of studies and Figure 3 shows the risk of bias of each item, as percentages.

###  Overview of outcome measures

 Among the included studies, 18 studies assessed the effectiveness of vanadium on GSH. In all studies, GSH level was significantly increased, except for one study,^46^ which observed no significant differences. Vanadyl sulfate supplementation significantly enhanced GSH levels in diabetes-induced animals.^12,17,18,39,42,49,50,52^ Other vanadium complexes such as oxovanadium (IV), Na[(O2)2(2,2’-bpy)] • 8 H2O, Na[VO(O2)2(1,10’-phen)] • 5 H2O, [VO(SO4)(1,10’-phen)] • 2 H2O, vanadyl (IV)-ascorbate (VOAsc), vanadium-3-hydroxy flavone, macrocyclic binuclear oxovanadium (IV) complex (MBOV), sodium orthovanadate, vanadium citrate, vanadium pentoxide, oxovanadium (IV) chelate also augmented GSH level.^2,13,14,24,37,43,51,59^

 Among the studies included, 27 studies analyzed the effectiveness of vanadium on GR, GST, and GPx levels. In one study, vanadium administration was inefficient in enhancing GPx level,^46^ and in five studies, vanadium administration significantly declined GPx levels.^16,42,53,54,63^ However, in the remaining of the studies, GPx level was significantly enhanced.^6,8,11,14,19-22,24,37,38,43,48,51,59,61^ Vanadium administration significantly increased GST level in 7 studies.^8,11,17,48,50,57,62^ In one study,^11^ no significant changes were observed. Vanadium administration significantly enhanced GR level in 4 studies.^8,11,19,24^ No significant changes were observed in one study,^42^ but a significant decline was observed in two studies.^53,54^

 In addition, 27 studies assessed the effect of vanadium on SOD level out of which 20 observed significant enhancement. Also, in 3 studies, significant alterations were not observed.^13,45,46^ In five studies, vanadium treatment declined SOD level.^16,42,47,53,54^ Vanadyl sulfate supplementation could also significantly augment SOD level.^2,21,41,42,44,48,50,53,54,60^ Other compounds such as oxovanadium (IV) complex, sodium orthovanadate, [VO(SO4)(1,10’-phen)] • 2 H2O, [VO(SO4)(2,2’-bpy)] • H2O, vanadyl(IV)-ascorbate, Dioxidovanadium (V) complex, vanadium-3-hydroxy flavone complex, macrocyclic binuclear oxovanadium (IV) complex, bis(maltolato)oxovanadium IV (BMOV), vanadium citrate, vanadium pentoxide also enhanced SOD level in different tissues of animals.^2,6,8,13,14,16,19,20,22,24,37,38,43,46,51,59,63^

 Among the included studies, 21 assessed the effect of vanadium on CAT levels. In all of the studies, the CAT level significantly increased, except for two studies in which vanadium administration was not efficient^8,46^ and in 4 studies in which CAT level declined.^16,42,45,52^ In 6 studies,^21,42,48,50,52,60^ vanadyl sulfate administration significantly restored the altered enzymatic activity level of CAT to normal level. Sodium orthovanadate, vanadium-3-hydroxy flavone complex, macrocyclic binuclear oxovanadium (IV) complex, bis(maltolato)oxovanadium (IV), vanadium citrate, oxovanadium (IV) chelate were also effective in restoring CAT enzymatic activity level to near normal level in different tissues.^6,14,16,19,21,22,24,37,45,46,51,59,63^ However, in one study sodium orthovanadate administration was not effective in altering CAT level.^8^

 Inflammatory biomarkers were assessed in 7 studies and significant decline was observed in all studies. Vanadyl sulfate supplementation significantly reduced TNF-α, IL-6, and hs-CRP.^40,41,44,58,63^ Vanadyl (IV)-ascorbate (VOAsc) complex and dioxidovanadium also reduced these inflammatory biomarkers.^43,61^ Caspase 3 level significantly decreased after oxovanadium (IV) complex and VOSO4 treatment.^66^

## Discussion

 In the present systematic review, most of the studies claimed beneficial features for vanadium concerning inflammatory and oxidative stress biomarkers in their overall conclusion, despite treatment with different compounds of vanadium, doses, species, methods of administration, and length of intervention. In the included studies, impaired enzymes levels expressed as either decrease or increase, were accepted as oxidative stress. For example, in few studies in which antioxidant enzymes were enhanced in the cells for compensating an antioxidant defense during diabetes, vanadium administration was able to restore the number of enzymes impaired.^42,53,54^ Despite the observed beneficial impact of vanadium compounds in controlling inflammatory and oxidative stress biomarkers, there were few studies that indicated no significant effects.

 In Saxena and colleagues’ study, vanadate supplementation was useful in optimizing antioxidant enzymes of diabetes-induced animal models. This was mainly because vanadium in the form of vanadate was able to create different forms of free radicals, or distract the antioxidant system.^46^ In Gupta and colleagues’ study, no significant changes in the activity of CAT was observed after vanadium treatment. In the study of Krośniak et al, vanadium (V) peroxocomplexes treatment was not efficient regarding SOD level. This may be due to various oxidative positions and organic/inorganic ligands of vanadium (V) peroxocomplexes.^13^ The increased NAD(P)H: quinone-oxidoreductase-1 activity in diabetic rats may have decreased the formation of ROS, which would explain why no changes in SOD level were observed.^45^ A recent study claimed that the administration of 1 mg/day BMOV in diabetes-induced animal models was not efficient in declining inflammatory biomarkers.^56^

 As far as we know, the current systematic review was the first to assess the effect of vanadium on inflammatory and oxidative stress biomarkers in diabetes-induced animals. The last publications regarding vanadium and diabetes were mainly regarding glycemic factors.^30^ Hence, several advantages could be mentioned for the present study. First, it included a high number of animal studies. Second, it assessed various outcomes. Third, it evaluated the effect of various organic and inorganic vanadium forms. Forth, it used the SYRCLE’s risk of bias tool for evaluating the methodological quality of studies. However, few limitations can be mentioned for this systematic review, as below: (1) non-English studies were excluded; (2) gray literatures were not extracted during further searches (3) studies that supplemented vanadium along with insulin and/ or other compounds were also included.

## Conclusion

 The present systematic review reaffirmed that vanadium compounds in different doses and methods of administration were efficient in normalizing inflammatory and oxidative stress biomarkers in T2DM. Furthermore, addressing high-quality clinical trials for assessing the effectiveness of vanadium is encouraged.

## Authors’ contributions

 FG and AO were involved in the concept of the manuscript; FG was responsible for writing the draft of the manuscript; MSA reviewed and edited the manuscript; and the final manuscript was read and approved by all of the authors.

## Funding

 The research protocol was funded by Student Research Committee, Tabriz University of Medical Sciences (grant number: 67836), Tabriz, Iran.

## Ethical approval

 Not applicable.

## Competing interests

 No conflict of interest was reported.

## Disclaimer

 The authors claim that no part of this paper is copied from other sources.

## Supplementary Files

Supplementary file 1 contains Table S1.
