# Supplementary material for: Vanadium and biomarkers of inflammation and oxidative stress in diabetes: A systematic review of animal studies
Source: Health Promot Perspect. 2022 Aug 20;12(2):122–30. doi: 10.34172/hpp.2022.16 (PMC9508397; doi:10.34172/hpp.2022.16)
Supplement: Supplementary file 1 — contains Table S1. [file hpp-12-122-s001.pdf]

Ghalichi et al, **Health Promotion Perspectives,** 2022, 12(2), 122-130
doi: 10.34172/hpp.2022.16
https://hpp.tbzmed.ac.ir

Vanadium and biomarkers of inflammation and oxidative stress in diabetes: A systematic review of animal studies

Faezeh Ghalichi[1], Alireza Ostadrahimi[2], Maryam Saghafi-Asl[3*]

[1]Student Research Committee, Tabriz University of Medical Sciences, Tabriz, Iran
[2]Department of Clinical Nutrition, Faculty of Nutrition and Food Sciences, Tabriz University of Medical Sciences, Tabriz, Iran
[3]Nutrition Research Center, Drug Applied Research Center, Department of Clinical Nutrition, Faculty of Nutrition & Food Sciences, Tabriz University of Medical Sciences, Tabriz, Iran

Table S1. Characteristics of studies included

Citation	Location	Randomization	Sample size	Population	Species	Mean weight (g)	Compound	Dosage	Method of administration	Length of intervention (day)	Outcome (Significant)
Sekar et al, 1990	India	N/A	(n=24)<br>Control=6<br>Vanadium-treated control=6<br>Diabetic=6<br>Vanadium-treated diabetic=6	Streptozotocin - induced diabetes	Male Wistar rats	160-180	SOV	0.3 mg/mL	Dissolved in drinking water, daily	15	↑ SOD, CAT, GPx and GSH levels in liver
Saxena et al, 1993	India	randomized	(n=24)<br>Control=6<br>Vanadium-treated control=6<br>Diabetic=6<br>Vanadium-treated diabetic=6	Alloxan monohydrate-induced diabetes	Male Wistar rats	200-220	SOV	0.6mg/mL	Dissolved in drinking water, daily	21	(-) GPx, SOD, CAT and GSH levels
Thompson & McNeil, 1993	Canada	randomized	(n=32)<br>Control=8<br>Vanadyl sulfate-treated control=8<br>Diabetic=8<br>Vanadyl sulfate-treated diabetic=8	Streptozotocin - induced diabetes	Male Wistar rats	N/A	Vanadyl sulfate	At first 0.5mg/mL and then gradually increased to 1 or 1.25 mg/mL	Dissolved in drinking water, daily	84	↑ Liver GST and GSH level in liver homogenates

Gupta and Baquer, 1998	India	N/A	(n=N/A)<br><br>Control<br><br>Diabetic<br><br>Insulin-treated diabetic<br><br>Vanadate-treated diabetic	Alloxan monohydrate-induced diabetes	Albino male rats of Wistar strain	180-200	SOV	0.6mg/mL	Dissolved in drinking water, daily	21	↑ SOD, GR, GPx and GST<br><br>(-) CAT
Genet et al, 2002	India	N/A	(n=60)<br><br>Control=12<br><br>Diabetic=12<br><br>Insulin-treated diabetic=12<br><br>Vanadate-treated diabetic=12<br><br>Trigonella-treated diabetic=12	Alloxan monohydrate-induced diabetes	Female Wistar strain rats	200-210	SOV	(first dose 0.2 mg/mL -second dose 0.6mg/mL)	Dissolved in drinking water, daily	21	↑ CAT, SOD, and GPx levels in heart, liver, kidney, and brain tissue
Gupta et al, 2004	India	N/A	(n=40)<br><br>Diabetic=10<br><br>Insulin-treated diabetic=10<br><br>Vanadate-treated diabetic=10<br><br>Vanadate-treated control=10	Alloxan monohydrate-induced diabetes	female rats of Wistar strain	180-200	SOV	0·2 mg/mL for 3 days, 0·4 mg/mL for another 3days, and 0·6 mg/mL for the rest of the intervention	Dissolved in drinking water, daily	21	↑ GR, GST and GPx levels in reticulocytes and red blood cell
Ramachandran et al, 2004	India	N/A	(n=24)<br><br>Control=6<br><br>MBOV-treated control=6<br><br>Diabetic=6<br><br>MBOV-treated diabetic=6	Streptozotocin - induced diabetes	Male albino Wistar rats	120-140	Macrocyclic Binuclear Oxovanadium (IV) complex (MBOV)	5 mg/kg body weight	Gavage, daily	30	↑ SOD, CAT, GPx, GSH, GST
Bolkent etal, 2005[1-7]	Turkey	randomized	(n=40)<br><br>Control=13<br><br>Vanadium-treated control =5<br><br>Diabetic=11<br><br>Vanadium-treated diabetic =11	Streptozotocin - induced diabetes	Male Swiss Albino rats	N/A	Vanadyl sulfate	100mg/kg	Gavage, daily	60	↑ GSH
Koyuturk et al, 2005	Turkey	randomized	(n=40)<br><br>Nondiabetic control=13<br><br>Vanadate-treated control=5<br><br>Diabetic=11<br><br>Vanadate-treated diabetic=11	Streptozotocin - induced diabetes	Swiss Albino male rats	N/A	Vanadyl sulfate	100mg/kg	Gavage, daily	60	↑ GSH level in Liver

Siddiqui et al, 2005	India	N/A	(n= 60)<br><br>Control=10<br><br>Diabetic=10<br><br>Insulin-treated diabetic=10<br><br>Diabetic treated with Trigonella=10<br><br>Vanadium-treated diabetic=10<br><br>Diabetic treated with Trigonella and Vanadium=10	Alloxan monohydrate-induced diabetes	Wistar female rats	200-220	SOV	0.6 mg/mL	Dissolved in drinking water, daily	21	↑ SOD and catalase<br><br>↓ GPx
R. Willsky et al, 2006	USA	N/A	(n=20)<br><br>Control=5<br><br>Vanadyl sulfate-treated control=5<br><br>Diabetic=5<br><br>Vanadyl sulfate-treated diabetic=5	Streptozotocin -induced diabetes	male wistar rats	N/A	Vanadyl sulfate	3 to 10 mM/Kg	Dissolved in drinking water, daily	28	↑ GST
Tunali and Yanardag, 2006	Turkey	randomized	(n=40)<br><br>Control=13<br><br>Vanadyl sulfate-treated control=5<br><br>Diabetic=11<br><br>Vanadyl sulfate-treated diabetic=11	Streptozotocin -induced diabetes	Swiss albino rats	N/A	Vanadyl sulfate	100 mg/kg/day	Gavage, daily	60	↑ Stomach and spleen GSH levels
Yanardag and Tunali, 2006	Turkey	randomized	(n=40)<br><br>Control=13<br><br>Vanadyl sulfate-treated control=5<br><br>Diabetic=11<br><br>Vanadyl sulfate-treated diabetic=11	Streptozotocin -induced diabetes	Male Swiss albino rats	N/A	Vanadyl sulfate	100 mg/kg	Gavage, daily	60	↓ Serum CAT<br><br>↑ Brain GSH level
Akgun-Dar et al, 2007	Turkey	randomized	(n=35)<br><br>Non-diabetic control=13<br><br>Vanadium-treated control=5<br><br>Diabetic=8<br><br>Vanadium-treated diabetic=9	Streptozotocin -induced diabetes	Male Swiss Albino rats	N/A	Vanadyl sulfate	100mg/kg	Gavage, daily	60	↑ Aorta GSH
Shukla et al, 2007	India	randomized	(n=5-8 in each group)<br><br>Normal	Streptozotocin -induced diabetes	male Balb/c mice	N/A	Bis(maltolato) Oxovanadium IV (BMOV)	0.2 mmol/kg	Dissolved in drinking water, daily	21	↓ CAT, SOD and GPx levels in liver and pancreas

			Vanadium-treated diabetic<br><br>Diabetic								
Ghareeb and Hussen, 2008	Egypt	N/A	(n=30)<br><br>Diabetic:<br><br>Untreated=10<br><br>Treated with sodium orthovanadate=10<br><br>Control=10	Alloxan monohydrate-induced diabetes	albino female rats	170	SOV	300mg/kg	Dissolved in drinking water, daily	45	↑ GST
Krośniak et al, 2009	Poland	N/A	(n=48)<br><br>Diabetic control=6<br><br>Insulin-treated diabetic=6<br><br>Insulin-treated diabetic+ Vanadium Complex1 (Di1)=6<br><br>Insulin-treated diabetic+ Vanadium Complex2 (Di2)=6<br><br>Insulin-treated diabetic+ Vanadium Complex3 (Di3)=6<br><br>Insulin-treated diabetic+ Vanadium Complex4 (Di4)=6<br><br>Insulin-treated diabetic+ Vanadium Complex5 (Di5)=6<br><br>Normal control=6	Streptozotocin - induced diabetes	Male<br><br>Wistar rats	220-250	1. Na((O2)2(2,2'-bpy)) • 8 H2O<br><br>2. Na(VO(O2)2(1,10'-phen)) • 5 H2O<br><br>3. Na(VO(O2)2(4,4'-Me2-2,2'-bpy)) • 8 H2O<br><br>4. (VO(SO4)(1,10'-phen)) • 2 H2O<br><br>5. (VO(SO4)(2,2'-bpy)) • H2O	50μmol/kg	Gavage, daily	56	↑ SOD<br><br>↑ GSH
Yanardag et al, 2009	Turkey	randomized	(n=21)<br><br>Control=5<br><br>(VOL)-treated control=5<br><br>Diabetic=6<br><br>(VOL)-treated diabetic=5	Streptozotocin - induced diabetes	male Swiss albino rats	N/A	Oxovanadium(IV) chelate (VOL)	0.2 mM/kg	Gavage, daily	12	↑ GSH, GPx levels and SOD levels in pancreas tissue<br><br>↓ CAT levels in pancreas tissue
Kurt et al, 2011	Turkey	randomized	(n=5-13 in each group)<br><br>Control<br><br>Vanadium-treated control<br><br>Diabetic<br><br>Vanadium-treated diabetic	Streptozotocin - induced diabetes	Male Swiss albino rats	N/A	Vanadyl sulfate	100 mg/kg	Gavage, daily	60	↓ SOD, CAT, GR, GPx, GST

Kumar et al, 2012	India	N/A	(n=36)<br><br>Control=6<br><br>Diabetic=6<br><br>Insulin-treated diabetic=6<br><br>Trigonella foenum graecum seed powder (TSP)-treated diabetic=6<br><br>sodium orthovanadate (SOV)-treated diabetic=6<br><br>Diabetic treated with TSP and SOV=6	Alloxan monohydrate-induced diabetes	Albino female rats of Wistar strain	180-200	SOV	0.6mg/mL	Dissolved in drinking water, daily	21	↑ SOD, CAT and GPx
Sanchez-Gonzalez et al, 2012	Spain	randomized	(n=37)<br><br>Control=9<br><br>Diabetic=8<br><br>Diabetic treated with 1mg V/d=10<br><br>Diabetic group treated with 3mg V/d=10	Streptozotocin - induced diabetes	Male Wistar rats	190–220	bis(maltolato)oxovanadium (IV) (BMOV)	1mg V/d<br><br>3mg V/d	Dissolved in drinking water, daily	35	↓ GSH-Px<br><br>(-) GR<br><br>↓ GST<br><br>↑ MDA
Tunali and Yanardag, 2013	Turkey	randomized	(n=40)<br><br>Control=13<br><br>Vanadyl sulfate-treated control=5<br><br>Diabetic=11<br><br>Vanadyl sulfate-treated diabetic=11	Streptozotocin - induced diabetes	Male swiss albino rats	N/A	Vanadyl sulfate	100 mg/kg/day	Gavage, daily	60	↑ Skin GSH levels and CAT, SOD and GST
Upreti et al, 2013	India	N/A	(n=36)<br><br>Control=6<br><br>Diabetic=6<br><br>insulin-treated diabetic=6<br><br>Vanadate-treated diabetic=6<br><br>A. indica-treated diabetic=6<br><br>diabetic treated with Vanadate and Azadirachta indica=6	Streptozotocin - induced diabetes	Albino male Wistar rats	200-210	SOV	0.2 mg/mL<br><br>and<br><br>0.6 mg/mL	Dissolved in drinking water, daily	21	↑ SOD, CAT, GR and GPx level in liver and kidney tissue<br><br>↑ GPx and GR level in kidney tissue
Pillai et al, 2014	India	N/A	(n=36)<br><br>Normal control=6<br><br>V3HF complex-treated normal=6	Streptozotocin - induced diabetes	Male albino Wistar rats	160-180	Vanadium-3-hydroxy flavone complex	5 mg/kg	Dissolved in drinking water, daily	30	↑ SOD, CAT, GSH and GPx, levels in pancreatic tissues

			3HF complex-treated normal=6<br><br>Diabetic=6<br><br>V3HF complex-treated diabetic=6<br><br>3HF complex-treated diabetic=6								
Sanchez-Gonzalez et al, 2014	Spain	randomized	(n=33)<br><br>Control=9<br><br>Diabetic=8<br><br>Diabetic treated with 6.2mg BMOV=8<br><br>High-dose diabetic group treated with 18.7mg BMOV=8	Streptozotocin -induced diabetes	Male Wistar rats	190-220	Bis(maltolato) Oxovanadium (IV)	6.2mg<br><br>and<br><br>18.7mg	Dissolved in drinking water, daily	35	↓ CAT<br><br>(-) SOD
Sun et al, 2014	China	randomized	(n=36)<br><br>Alloxan-treated=6<br><br>0.45SV-treated=6<br><br>0.9SV-treated=6<br><br>1.8SV-treated=6<br><br>Control group=6	Alloxan monohydrate-induced diabetes	Wistar rats	150-200	Sodium Vanadate	0.05 mmol/kg body weight<br><br>0.1 mmol/kg body weight<br><br>0.2 mmol/kg body weight	Dissolved in drinking water, daily	45	↓ SOD (insignificant)
Xie et al, 2014	China	randomized	(n=42)<br><br>Diabetic=7<br><br>H2dipic-Cl treated=5<br><br>V3dipic-Cl treated=5<br><br>V4dipic-Cl treated=5<br><br>V5dipic-Cl treated=5<br><br>VOSO4 treated=5<br><br>NaVO3 treated= 5<br><br>Control= 5	Streptozotocin -induced diabetes	male wistar rats	230-250	H2dipic-Cl<br><br>V3dipic-Cl<br><br>V4dipic-Cl<br><br>V5dipic-Cl<br><br>VOSO4<br><br>NaVO3	50 µgV/ml	Dissolved in drinking water, daily	28	↑ CAT, SOD and GPx levels in liver tissue<br><br>↑ GSPx
Yilmaz-Ozden et al, 2014	Turkey	randomized	(n=40)<br><br>Control=13<br><br>Vanadyl sulfate-treated control=5<br><br>Diabetic=11<br><br>Vanadyl sulfate-treated diabetic=11	Streptozotocin -induced diabetes	Male Swiss albino rats	N/A	Vanadyl sulfate	100 mg/kg	Gavage, daily	60	↓ GR, GPx, GST

Yilmaz-Ozden et al, 2014	Turkey	randomized	(n=40)<br><br>Control=13<br><br>Vanadyl sulfate-treated control=5<br><br>Diabetic=11<br><br>Vanadyl sulfate-treated diabetic=11	Streptozotocin - induced diabetes	Male Swiss albino rats	N/A	Vanadyl sulfate	100 mg/kg	Gavage, daily	60	↓ GPx, GST and SOD levels in lung tissue
Liu et al, 2015	China	randomized	(n=40)<br><br>High-fat low-sucrose diet (HFSD)=10<br><br>HFSD supplemented with Rosiglitazone=10<br><br>HFSD supplemented with sodium L-ascorbate=10<br><br>HFSD supplemented with Vanadyl (IV)-ascorbate (VOAsc) complex=10	High-fat low-sucrose diet for inducing insulin resistance	Male C57BL/6J mice	18-20	(VOAsc) complex	0.45mg/kg	Gavage, daily	48	↓ TNF-α<br><br>↑ SOD, GPx, GSH, CAT
El-Karib et al, 2016	Saudi Arabia	randomized	(n=36)<br><br>Control=6<br><br>Vanadium-treated control=6<br><br>Diabetic=6<br><br>Insulin-treated diabetic=6<br><br>Vanadium-treated diabetic=6<br><br>Insulin and Vanadium-treated diabetic=6	Streptozotocin - induced diabetes	Male Sprague Dawley rats	200-250	Vanadyl sulfate	0.64 mmol/kg	Dissolved in drinking water, nasopharyngeal tube, daily	56	↓ IL-6 and TNF-α<br><br>↑ SOD
Bin-Jaliah et al, 2018	Saudi Arabia	N/A	(n=60)<br><br>Control=10<br><br>Vanadium-treated control=10<br><br>High fat diet-treated diabetic =10<br><br>Insulin-treated diabetic =10<br><br>Vanadium-treated diabetic=10	Streptozotocin - induced diabetes	Male Sprague Dawley rats	150-200	Vanadyl sulfate	0.64 mmol/kg	Dissolved in drinking water, esophageal tube, daily	56	↓ TNF-α, IL-6, and hs-CRP
Samira et al, 2018	Tunisia	N/A	(n=60)<br><br>Control=10	Streptozotocin - induced diabetes	Male Wistar rats	175-200	Vanadyl sulfate	5 mg/kg body weight<br><br>and	Intraperitoneal injection, daily	30	↑ SOD and CAT

			Control treated with 5 mg/kg VOSO4=10<br><br>Control treated with 10 mg/kg VOSO4=10<br><br>Diabetic=10<br><br>Diabetic treated with 5 mg/kg VOSO4=10<br><br>Diabetic treated with 10 mg/kg VOSO4=10					10 mg/kg body weight			
Sushko et al, 2018	Ukraine	N/A	(n=40)<br><br>Control=8<br><br>Diabetic=8<br><br>Diabetic treated with 0.125 µg/mL Vanadium citrate=8<br><br>Diabetic treated with 0. 5 µg/mL Vanadium citrate=8<br><br>Diabetic treated with 2.0 µg/mL Vanadium citrate=8	Alloxan monohydrate-induced diabetes	Laboratory rat	100-120	Vanadium citrate	0.125 µg/mL<br><br>0.5 µg/mL<br><br>2.0 µg/mL	Dissolved in drinking water, daily	30	↑ SOD, CAT, GPX, GR and GSH levels in pancreas
Morsy et al, 2019	Egypt	randomized	(n=50)<br><br>Control=10<br><br>Vanadium-treated control=10<br><br>Diabetic=10<br><br>Insulin-treated diabetic=10<br><br>Insulin and Vanadium-treated diabetic=10	Streptozotocin - induced diabetes	Male Sprague-Dawley rats	150-200	Vanadyl sulfate	0.64 mmol/kg	Dissolved in drinking water, esophageal tube, daily	42	↓ TNF-α and IL-6<br><br>↑ Hepatic SOD level<br><br>↓ serum hs-CRP levels
Sibiya et al, 2019	South Africa	N/A	(n=24)<br><br>Diabetic=6<br><br>Diabetic treated with 10 mg/kg Dioxidovanadium=6<br><br>Diabetic treated with 20mg/kg Dioxidovanadium=6<br><br>Diabetic treated with 40mg/kg Dioxidovanadium=6<br><br>Insulin-treated diabetic=6	Streptozotocin - induced diabetes	Sprague-Dawley rats	240-250g	Dioxidovanadium	10, 20, 40 mg/kg	gavage needle, twice every 3rd day	35	↓ GPx and hs-CRP
Vijay et al, 2019	India	randomized	(n=40)	Streptozotocin - induced diabetes	adult male wistar rats	150-200	Vanadium pentoxide	5 mg/kg body weight	Gavage, daily	28	Vanadium pentoxide nanoparticles:<br><br>↑ CAT, SOD, GSH and GPx

			Untreated normal control=10<br><br>Diabetic control=10<br><br>Diabetic treated with 5 mg/kg body weight Vanadium pentoxide=10<br><br>Diabetic treated with 5 mg/kg body weight Vanadium pentoxide nanoparticles=10				Vanadium pentoxide nanoparticles				
Bin-Jaliah et al, 2020	Saudi Arabia	randomized	(n=24)<br><br>Control=6<br><br>Vanadium-treated control=6<br><br>High fat diet-treated diabetic=6<br><br>Vanadium-treated diabetic=6	Streptozotocin - induced diabetes	Male Sprague Dawley rats	150-200	Vanadyl sulfate	0.64 mmol/kg	Dissolved in drinking water, esophageal tube, daily	56	↓ TNF-α and IL-6
El-Shafey and Elsherbiny, 2020	Egypt	N/A	(n=28)<br><br>Normal control=15<br><br>Normal-Vanadium complex treated=15<br><br>Normal-VOSO4 treated=15<br><br>Diabetic untreated=15<br><br>Diabetic Vanadium complex treated=15<br><br>Diabetic-VOSO4 treated=15	Streptozotocin-induced diabetes	Male Albino mice	24-33	Vanadyl sulfate (VOSO4)<br><br>&<br><br>Oxovanadium (IV) complex	N/A	N/A	19	Oxovanadium complex:<br><br>↑ GSH<br><br>↑ SOD<br><br>↓ Caspase 3<br><br>VOSO4:<br><br>↑ SOD<br><br>↓ Caspase 3
Mbatha et al, 2020	South Africa	N/A	(n=24)<br><br>Non-diabetic control=6<br><br>Diabetic control=6<br><br>Insulin-treated diabetic=6<br><br>Vanadium complex-treated diabetic=6	Streptozotocin - induced diabetes	Male Sprague–Dawley rats	N/A	Dioxidovanadium (V) complex,<br><br>Cis(VO2(obz)py))	40.0 mg/kg	gavage needle, twice every 3rd day	20	↓ MDA<br><br>↑ SOD and GPx<br><br>↓ hsCRP level in heart and plasma
Tunali et al, 2020	Turkey	randomized	(n=40)<br><br>Untreated, nondiabetic controls=13	Streptozotocin - induced diabetes	Male rats	N/A	Vanadyl sulfate	100 mg/kg/day	Gavage, daily	60	↑ CAT, SOD, GPx and GST in liver tissue

			Vanadyl sulfate-treated control=5<br><br>Diabetic=11<br><br>Vanadyl sulfate-treated diabetic=11								
Sánchez-González et al, 2021	Spain	randomized	(n=29)<br><br>Control=9<br><br>Diabetic=10<br><br>Diabetic treated with 1 mg V/day=10	Streptozotocin - induced diabetes	Male Wistar rats	190–220	bis(maltolato)oxovanadium(IV) (BMOV)	1 mg V/day	Dissolved in drinking water	35	(-) CRP<br><br>(-) IL-6<br><br>(-) IL-1β<br><br>(-) TNF-α

Tumour Necrosis Factor alpha (TNF-α), Interleukin 6 (IL-6), high-sensitivity C-reactive protein (hs-CRP), Glutathione (GSH), Superoxide dismutase (SOD), Catalase (CAT), Glutathione peroxidase (GPx), Glutathione S-transferase (GST), Glutathione reductase (GR), Not applicable (N/A); sodium orthovanadate (SOV)
